# Hotspots of H1N1 influenza in India: analysis of reported cases and deaths (2010–2017)

**DOI:** 10.1177/0049475519879357

**Published:** 2019-11-26

**Authors:** Pranab Chatterjee, Bhavna Seth, Tamoghna Biswas

**Affiliations:** 1 Public Health Specialist, Translational Global Health Policy Research Cell, Indian Council of Medical Research Scientist B, Indian Council of Medical Research, National Institute of Cholera and Enteric Diseases, Kolkata, India; 2 Fellow, Department of Pulmonary and Critical Care Medicine, Johns Hopkins University, Baltimore, MD, USA; 3 Pediatrics Consultant and Independent Researcher, Kolkata, India

**Keywords:** H1N1, influenza hotspots, pandemic, India

## Abstract

Influenza A (H1N1) caused significant mortality and morbidity globally. We identified the hotspots for H1N1 influenza in India using cases and deaths reported in the Integrated Disease Surveillance Program between 2010 and 2017. A total of 114,667 cases and 8543 deaths were reported from across India, at an overall case fatality rate of 7.5%. While Maharashtra accounted for 21% of cases and 31% of deaths, Delhi and Gujarat were ranked the highest based on the population-adjusted ranks for morbidity and mortality, respectively. The current analysis identified states and union territories in western India (Delhi, Punjab, Rajasthan, Gujarat and Maharashtra) to be especially vulnerable.

## Introduction

H1N1, which reached pandemic status in June 2009,^1^ also affected India, a recognised hotspot for emerging infectious diseases (EIDs). We analysed publicly available data on H1N1 from the Integrated Disease Surveillance Program (IDSP), the national disease surveillance and monitoring program of India, to identify the reported hotspots of H1N1 outbreaks.^2^

## Materials and methods

H1N1 cases were reported to the IDSP from all 36 states and union territories (S/UTs) through a network of laboratories from 2010 to 2017. These data are available in the public domain through the IDSP. We undertook a descriptive analysis to identify the burden of the disease across different states and computed the case fatality rates (CFRs). Based on the population reported in the 2011 census, we reported the number of cases and deaths per 100,000 people.^3^ Based on this population-adjusted value, each S/UT was given a rank for the reported cases and deaths for each year between 2010 and 2017. Two average ranks for the state, for reported cases and reported deaths were computed and the mean of these two average ranks was calculated to create an index rank representative of the burden of H1N1 in the S/UTs. States with higher estimates received a higher rank; thus, a state with a higher burden would have a higher rank, indicated by a smaller numerical value. For S/UTs which had tied ranks, the mean rank (mr) was accorded to all tying members.

## Results

Between 2010 and 2017, there were a reported 114,667 cases and 8543 deaths due to H1N1from India, at an overall CFR of 7.5%. While Maharashtra accounted for 21% of cases (n = 23,812) and 31% of deaths (n = 2648), Delhi (mr = 3; total cases = 11,703) and Gujarat (mr = 3.75; total deaths = 1651) were ranked the highest based on the population-adjusted ranks for morbidity and mortality, respectively.

The five top-ranked S/UTs for reported cases—Delhi (mr = 3), Telangana (mr = 4.75), Gujarat (mr = 6.38), Karnataka (mr = 6.75) and Goa (mr = 7.38)—accounted for 41% of all cases of H1N1 in India. The five S/UTs to report the greatest number of cases—Maharashtra, Gujarat, Rajasthan, Delhi and Karnataka—accounted for 68% of all H1N1 cases reported in India.

The five top-ranked S/UTs for reported deaths—Gujarat (mr = 3.75), Rajasthan (mr = 4.75), Maharashtra (mr = 5.75), Punjab (mr = 8) and Kerala (mr = 9.75)—accounted for 71% of all deaths from H1N1 in India. The five S/UTs to report the greatest number of deaths—Maharashtra, Gujarat, Rajasthan, Madhya Pradesh and Karnataka—accounted for 76% of all H1N1 deaths reported in India. The four states that reported the highest numbers of deaths also recorded a higher-than-national average CFR (Maharashtra = 11.1%, Gujarat = 9.1%, Rajasthan = 8.9%, Madhya Pradesh = 17.7%).

**Figure 1.**
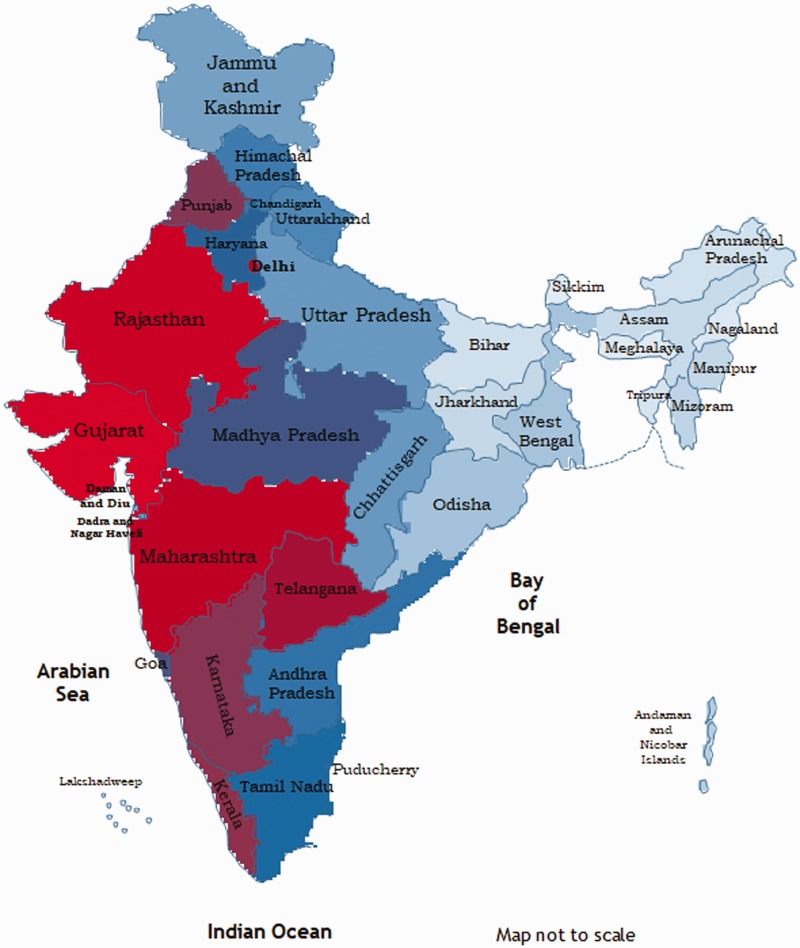
Heat map of India showing States and Union Territories of India vulnerable to H1N1 influenza. States and Union Territories which have a lower rank, and hence a higher vulnerability to H1N1 influenza, have been shown in red, and those with a higher rank, indicating a lower vulnerability to H1N1 influenza have been shown in light blue. States and Union Territories with intermediate vulnerability has been shown in decreasing shades of blue. Vulnerability was computed as the average of the mean rank for reported cases and reported deaths over the eight-year period (2010–2017).

In this eight-year period, Lakshadweep and Sikkim reported no cases of H1N1. The eight states of North East India (Arunachal Pradesh, Assam, Nagaland, Mizoram, Meghalaya, Tripura, Manipur and Sikkim) cumulatively accounted for only 326 cases (0.3%) and 15 deaths (0.2%), with a majority being reported from Assam (234 cases and 10 deaths). This indicates a possibility that the reported numbers underestimate the magnitude of the actual problem. However, these data do show the presence of hotspots of vulnerability to H1N1 in India. When a heat map was created from the average of the mean ranks for cases and deaths from H1N1 over the past eight years (Figure 1), S/UTs along the western border of the nation— Punjab, Rajasthan, Gujarat, Maharashtra and Delhi—showed higher vulnerability compared to the other S/UTs of the nation.

## Discussion

It is apparent that there are slight deviations in the heat map generated based on the current analysis than the ones generated using the burden of lower respiratory infections estimated by the India State-Level Disease Burden Initiative.^4^ This discrepancy may be explained by the difference in laboratory capacity across various regions in diagnosing H1N1 using polymerase chain reaction (PCR) technology. In addition, the capacity of providing healthcare services and access to diagnostic and therapeutic facilities, as well as efficiency of reporting mechanisms for cases to the IDSP is also likely to vary across S/UTs, affecting these estimates. Finally, as recent evidence has shown, other types of influenza (like type B, H3N2) also circulate in India, often dominating seasonal trends, thus possibly resulting in the slight differences from the hotspots identified previously.^5^

The continuing spate of cases and deaths from H1N1 demands that a renewed focus be accorded to this issue. Vaccination in susceptible populations needs to be explored as a potential public health response. Strengthening of surveillance to improve reported estimates is a priority as it would advise the process of investing in the public health response to H1N1 in India.
